# Bird–Borrelia Interactions: A Historical Review and Their Significance for Human Disease Ecology

**DOI:** 10.3390/microorganisms14051096

**Published:** 2026-05-12

**Authors:** András P. Bózsik, Dömötör M. László, Borisz Egri

**Affiliations:** 1 Kázmér Albert Department, Széchenyi István University, 9026 Győr, Hungary; apbozsik@gmail.com; 2 Lyme Diagnostics Ltd., 2011 Budakalász, Hungary

**Keywords:** avian, migratory birds, *Borrelia anserina*, *Borrelia garinii*, ticks, reservoir, epidemiology

## Abstract

Research increasingly identifies wild birds, particularly long-distance migratory species, as epidemiologically relevant hosts and vectors for tick-borne *Borrelia* species that pose risks to both avian and human health. This review contextualizes avian-associated *Borrelia* research historically and microbiologically, showing the role of avian hosts in the ecology of agents causing relapsing fever and Lyme borreliosis. We identify key publications that trace the evolution of *Borrelia* research—from early microscopic observations of spirochetes to the modern molecular and serological evidence. The review collects literature on the process by which *Borrelia* gained early scientific attention due to its characteristic morphology and elevated bloodstream concentrations during septicemic phases, which enabled early etiological links between the microbe and disease. It follows the recognition of avian spirochetosis caused by *Borrelia anserina* and charts the shift in focus after the discovery of *Borrelia burgdorferi* sensu lato (Subgen. novum recomm. Borreliella, Lyme-group *Borrelia*). Publications listed show that birds can transport infected human-parasitic ticks over long distances and, in certain bird species, selectively amplify Lyme-group *Borrelia* species, especially *Borrelia garinii*, which has the highest temperature tolerance and is thus potentially viable in avian hosts. The literature supports the role of birds in maintaining and disseminating *Borrelia* infections and infected ticks across continents.

## 1. Introduction

According to the NCBI Taxonomy database, and the Genome Taxonomy Database (GTDB), the family Borreliaceae contains two pathogenic genera: *Borrelia* (containing relapsing-fever-type *Borrelia*) and *Borreliella* (containing *Borrelia*-causing Lyme borreliosis, formerly known as *Borrelia burgdorferi* sensu lato, Bbsl). However, a taxonomic publication has proposed that one single genus, *Borrelia* Swellengrebel 1907, should encompass all these species [1]. To clarify genetic categorization and avoid confusion, scientists at leading *Borrelia* research centers have proposed a new consensus that the single *Borrelia* genus should consist of two subgenera: *Borrelia* and *Borreliella* [2]. This proposal has not yet gained widespread acceptance, and for clarity in this work, we will use the original (pre-2013) terms for the two subgenera, “relapsing-fever-type *Borrelia*” and “*Borrelia burgdorferi* sensu lato” (Bbsl), when referring to the genetic variants or species of the *Borrelia* genus.

*Borrelia* genus bacteria are 0.2–0.3 µm wide and 10–30 µm long with a flat-wave morphology. Periplasmic flagella inserted at the tips overlap in the central region to provide motility. The bacteria grow in a complex, serum-supplemented liquid medium and are microaerophiles, and ideal growth temperatures are 33–37 °C. Vectors are ticks of the genus *Ixodes* for Bbsl and argasid ticks, ixodid ticks, or the human body louse for relapsing-fever-type *Borrelia*. Their genome consists of a linear chromosome and linear and circular plasmids [2].

Wild birds, especially migratory ones, have recently been studied as carriers or potential reservoirs for tick-borne infections, particularly various *Borrelia* species. These include human and avian pathogens, for which wild birds are gradually recognized as an epidemiological factor [3,4,5]. In this paper, we review literature on the main stages of research concerning avian and human-pathogenic species of *Borrelia* in wild birds and birds of prey, and compile studies examining their epidemiological relevance to humans.

The historical scientific relevance of *Borrelia* species lies in their characteristic shape and movement pattern, and in their ease of observation due to the relatively high concentration of relapsing-fever-type *Borrelia* during septicemic periods, which align with severe symptoms. For this reason, *Borrelia* was among the first microorganisms to be studied under a microscope and identified as a disease-causing agent.

We will draft the process leading to the discovery that wild birds can be infected by the avian pathogen *B. anserina* and develop lifelong immunity. While wild birds can transfer infected human-parasitic ticks across continents or globally, they can also serve as reservoirs of human-pathogenic Bbsl (subgen. novum recomm. *Borreliella*) and can carry or amplify the bacteria. Publications also show that Bbsl is present in various tissues of birds, causes mild symptoms, and elicits an immune response. However, this immune response does not prevent reactivation or reinfection. This is why wild birds are suspected of spreading the causative agent of Lyme borreliosis worldwide. The role of migratory birds in the spread of tick-borne diseases has long been emphasized. Yet the biological and physiological relationships between different bird species, ticks, and pathogens are not yet fully understood. It is important to monitor the presence and the behavior not only of ticks, but also of their hosts, given dynamic changes in climatic and environmental conditions and, consequently, the migration and breeding seasons of migratory birds carrying ticks and tick-borne pathogens. This may explain how tick-borne zoonotic diseases are diagnosed in new environments distant from known ones [6].

The objective of this review is to examine the long history and interconnection between migratory birds and their *Borrelia* infections, how the investigation of wild birds’ infections contributed to the science of human diseases, and to list the growing number of sources that underpin the human epidemiological relevance of wild birds in *Borrelia* infections. We will concentrate the review especially on the large-bodied migratory birds of Europe. However, important discovery steps relevant to the topic, made in other areas of the Northern Hemisphere and/or in other migratory wild bird species, will be mentioned whenever they constitute a first demonstration or the demonstration of a topic that has not yet been fully investigated in large-bodied migratory birds.

### 1.1. Identification and Scientific Importance

Because of their distinctive shape and movement, as well as their larger-than-average length, spirochete bacteria (Spirochaetia, Paster 2020) were among the first to be identified (*B. anserina*, *Treponema* species). Dutch naturalist Antonie van Leeuwenhoek discovered spirochetes through microscopic investigation in 1683. A *Borrelia* genus, possibly *Borrelia recurrentis*, was identified by Obermeier in 1867 (published in 1869 and 1873) as the cause of a severe human infection [7]. This was the first microbiological proof of a disease, observed 11 years before Robert Koch’s discovery, which supported Kircher’s “germ theory”.

The potential presence of spirochetoid forms may date back to the early days of bacterial life on the planet [8], whereas the first genetic evidence for *Borrelia* is from 5300 years ago, where the symptoms caused by *Borrelia burgdorferi* could also be observed, besides the presence of parts of its genome [9]. Nevertheless, the road to connecting *Borrelia* and the symptoms has not always been so direct.

### 1.2. The Indirect Proof of Borrelia Presence via Symptoms

In the early days of medicine, deer and ticks were linked to the spreading of *Borrelia*-specific symptoms. Ancient Chinese medicine described “deer disease” (Song dynasty), depicting the symptoms of Lyme disease, brain “Gu syndrome” (Qugu Ranxi Lu, Qing Dynasty Daoist doctor) [10,11], the symptoms of which align with Lyme neuroborreliosis (e.g., a tick bite), and “Bi syndrome”, which includes a variety of symptoms, including Lyme arthritis [12].

In the Middle Ages, according to the research of Drymon (2008) [13], the appearance of the so-called “witch marks” was geographically concurrent with evidence of Lyme disease, the marks resembling the Lyme bullseye rash, and neurological symptoms being recorded in the persons bearing the marks.

Reverend Walker, while visiting Jura (commonly known as “Deer Island”) in Scotland in 1764, described how a tick bite caused symptoms of Lyme disease. It is important to note that the island remains known today for its abundant birdlife, especially birds of prey like golden and white-tailed eagles, and for its large red deer populations. This serves as a link, indicating that the island could be infested with *Borrelia*-infected ticks via the birds, and that the infection cycle can be maintained by the deer population, along with small rodents and rabbits that serve as prey for the eagles.

Beard (1869) described neurasthenia, a description closely resembling neuroborreliosis [14].

Buchwald (1883) first described the chronic skin rash, or erythema migrans, the sign of what is now known to be Lyme disease [15].

### 1.3. The Direct Study of Borrelia Spirochetes

The morphological study of *Borrelia* bacteria was aided by the invention and advancement of the microscope, especially during the 19th century by scientists such as Lister, Zeiss, Abbé, Otto, Koenig, and Koehler. Early on, the terms used for *Borrelia* were vague, such as Spirillum and Spirochetes, which often described various *Borrelia* species in different hosts. Especially given the lack of credible visual evidence, it is hard to distinguish which examination identified which species of causative agent (and/or artifacts). Nevertheless, we can conclude that the early investigations identified the relapsing-fever type of *Borrelia*, since they cause a more fulminant disease in both humans and animals, and they are easier to detect, as they are 10–1000 times more concentrated in the blood than Bbsl [16]. Furthermore, the *Borrelia* found in bird hosts can be concluded to be *B. anserina*, also from the relapsing-fever-type subgenus, for the same reason, because the presence of human-pathogenic Bbsl in birds could only be proven with methods of lower limits of detection like PCR or microscopy based on concentration [17].

Spirochetes were extensively studied from the first half of the 19th century onward, including Ehrenberg (1835), who coined the term Spirochete. The first identifications came from samples of birds. Kent identified a spirochete from the intestine of birds in 1880, *Spirochaeta eberthi*. Sakharov identified in 1891 that *Spirochaeta anserina* caused septicemia in various genera of birds, while Blanchard identified in 1905 that *Spirochaeta gallinarum* caused septicemia among chickens [18].

Regarding the detailed study of spirochetes, Schaudinn and Hoffman (1905) first presented the lifecycle of a spirochete, *Treponema pallidum* [19].

Dutton et al. (1905), and independently Koch (1905), identified that ticks can carry and transmit the *Borrelia* bacteria, and also observed transovarial transmission, when researching evidence of the outbreak of a recurrent fever epidemic from 1857 in Africa [20].

Prowazek (1909) wrote the “Contribution to the study of the development of *Spirochaeta gallinarum*”, published in the Portuguese language, which is a seminal work that details the morphology, lifecycle, and pathogenesis of the spirochete *S. gallinarum* [21].

The lifecycles of *S. duttoni* and, importantly, *S. gallinarum* were described in detail by Hindle (1911) [22], who also gave a comprehensive account of the previous publications. He describes the lifecycle from the spreading by *Argas persicus* ticks, the various forms during the course of the infection, and the disease in birds [22]. Balfour also presented similar results in his articles and presentations at the same time. Based on these exact descriptions, one can already suspect that *S. gallinarum* was actually the current-day *B. anserina*.

Amadée Borrel studied the morphology of spirochetes for several years and showed that *S. gallinarum* was similar to the spirochete identified by Obermeier in 1867 that was associated with relapsing fever (*B. recurrentis*) in Berlin. *S. gallinarum* was probably identical to *Spirillum anseri*, identified by Sakharroff in diseased geese in 1891 [23], given that the morphological differences between the two descriptions of spirochetes can easily be explained by the changes caused by the number of passages on hosts, or the number of generations in cultivation.

### 1.4. Birds as Potential Epidemiological Factors

The presence of *Borrelia* in birds and the fact that human-parasitic ticks can attach to birds suggest that birds may be potential factors in the spread of human diseases. Without giving a full review of the connection between ticks and birds here, Pavlovsky (1940) first proposed the scientific theory that birds facilitate the spread of tick-borne diseases [24], then his academic disciple supported this theory by evidence [25], and later Korenberg (1962) confirmed it in natural foci [26]. It has also been suggested that the long-range movements of migratory birds might result in transportation of infected ticks to previously uninfected areas: Hoogstraal et al. (1961, 1963) [27,28].

A relatively thorough description is given by Sixl and Nosek (1972) of the kind of diseases spread by ticks, including Borrelial infections and their hosts, including passeriform birds [29], even before the identification of *Borrelia burgdorferi* as the causative agent of Lyme borreliosis.

### 1.5. Borrelia Spirochetes in Birds

In a study, Novy (1906) [30] reported on his own and other researchers’ observations of spirochete-caused diseases. He mentioned Sacharoff’s (1890) note, in which he had said that *Sp. anserina* causes recurrent fevers in geese, and that ducks and chickens can also become infected by injecting infected blood. He also mentioned the discovery of two other researchers, Marchoux and Salimbeni, in 1903, about the observation that the tick-borne *Sp. gallinarum* results in similar symptoms in chickens [30], potentially caused by the ticks *Argas persicus*.

In probably the most detailed work on the avian pathogen, McNeil et al. (1949) described the history of *B. anserina* [31], focusing on the American continent, collected many case reports from 1920 to 1948, and showed that the infection is host-restricted in birds. They provided a complete morphological description, cultivation methods, worldwide epidemiology, and details about the course of infection and necropsy results. The article includes treatment options and experience and describes their own field experiences, including the fact that wild birds were not infected around a major outbreak of infection at a turkey farm.

Felsenfeld (1971) published his book on “*Borrelia*: strains, vectors, human and animal borreliosis”, a 180-page book with 749 references, making a complete account of what can be known about borreliosis [32].

Prudovsky et al. (1978) described the previous attempts to identify *B. anserina* by immunological methods and defined the indirect fluorescent antibody method [33].

By 1979, DaMassa and Adler had published a large number of articles on the virulence of *B. anserina* in various poultry with different immunocompetence [34,35].

The excellent efficacy of antibiotic treatment, the relatively low genetic variation of *B. anserina*, the lifelong adequate immune protection in surviving animals, and changes in poultry breeding methods all contributed to the successful eradication of avian borreliosis from many parts of the world. The research knowledge remained, and even became a little dormant, until a new disease emerged, in which the causative *Borrelia* possess high genetic variability, are less susceptible to antibiotics, and there is no lifelong protection against them.

### 1.6. “Borrelia Research” Intensifies After the Description of Lyme Borreliosis

*More focus was directed toward all Borrelia species after the identification of Bbsl as the causative agent of Lyme borreliosis.*

In 1984, a new Lyme disease hotspot emerged in New Jersey, with human and canine infections likely introduced by ticks carried by birds and rodents. The realization that a previously uninfected area with a radius of nearly 5 km had become the starting point for the spread of the bacterium in less than a year may have drawn more attention to the *Borrelia* ecosystem [36].

Infection of wild birds by human-pathogenic *Borrelia* (besides *B. anserina*) was detected by Anderson and Magnarelli (1984) [37], although confirmation of *Borrelia* presence in the cultivated blood sample was performed only by dark-field microscopy. Specific examination confirmed the presence of *B. burgdorferi* in the ticks feeding on the birds. It was already concluded in 1986 in a paper by Anderson et al. [38] that wild birds can be infected by *B. anserina* and by the human-pathogenic Bbsl and can serve as the means of “cosmopolitan distribution” for *Borrelia* species, but it took almost two decades for this concept to be widely adopted.

Barbour and Hayes (1986) published their summary of “Biology of *Borrelia* Species”, which, however, does not go into detail on the role of avian hosts apart from mentioning *B. anserina* [39].

Over the following decade, many studies were carried out to identify tick species parasitizing birds and the pathogenic bacteria they harbor, as well as to obtain a more accurate picture of the interspecies relationships involved in transmission.

After the controversy over field and experimental data, by the 2000s it had become a generally accepted position in research on *Borrelia* species that birds play a significant role in the spread of *B. anserina* and Bbsl in Asia, Europe, and North America. Not only terrestrial birds, but also waterfowl and migratory birds can create new foci of the disease during their lives and transmit spirochetes to ticks that have not yet come into contact with the bacterium [40].

After its discovery and intense research for decades, *B. anserina* was for a certain period considered a rare or “forgotten” disease, while Faccini-Martínez et al. conclude in 2022 that its real geographical distribution is vastly underestimated due to misdiagnosis of symptoms (e.g., confusion with malaria) and the fact that there is no easy laboratory diagnostic method for the afebrile periods of relapsing-fever-type *Borrelia* infections [4]. Furthermore, it can be concluded that migratory birds can strongly influence their spreading [16].

## 2. Modern-Day History of *Borrelia* Investigations in Birds, with a Focus on Wild Birds

### 2.1. Research on Avian-Pathogenic Borrelia Infections

*Research on B. anserina intensified and the identification of other new avian-specific Borrelia followed.*

Levine et al. (1990) provided evidence that is important for *Borrelia* research, namely, that *B. anserina* loses its infectivity after 12 serial passages in Barbour–Stoenner–Kelly (BSK) medium, whereas no morphological differences were observed, only changes in the protein profile [41].

Fabbi et al. (1995) tested a serological reaction in 3186 urban pigeons (*Columba livia livia*) collected in Italy, out of which 104 gave a positive seroreaction to *B. anserina* and most probably not to *B. burgdorferi* [42]. Pigeons are known to have very high body temperature (43 +/− 0.5 °C) and, therefore, they have a higher chance of resisting human-pathogenic *Borrelia*, whereas *B. anserina* has a higher heat tolerance, and the surviving pigeons will develop a lifetime immune response against the infection.

Hamer et al. (2012) [43] demonstrated the role of wild birds in the maintenance and dissemination of vectors and microbes by collecting ticks from almost 20,000 wild birds and investigating them for *Borrelia* using nested PCR. Here, 86.5% of the 12,432 ticks collected were *Ixodes dentatus*, a tick that specializes on birds and rabbits, and only <1% were *I. scapularis*. The *Borrelia* infectivity rates of ticks were relatively low for the two special *Borrelia* tested, Candidatus *Borrelia andersoni* (Marconi 1995) (1.6%), a serovariant specialized to birds, and the relapsing-fever-type *Borrelia*, *B. miyamotoi* (0.7%), transmitted by hard ticks (hard-tick-borne relapsing fever, HTBRF) specifically belonging to the *Ixodes* genus, the same genus that transmits Bbsl. They claim this as the first identification (at least in the US) of *B. miyamotoi* [43].

Cordeiro et al. (2022) presented a novel enzyme-linked immunosorbent assay (ELISA) for the detection of IgY antibodies against *B. anserina* in hen (*Gallus gallus domesticus*) [44].

### 2.2. Research on Bird-Collected Ticks Carrying Human-Pathogenic Infections, Without the Demonstration of Reservoir Competence

*Birds carry various species of ticks, many of which are specialized to birds, others are generalists, and some are specialized to humans. The first step toward a potential epidemiological contribution is to determine whether these ticks are infected with human-pathogenic Borrelia.*

Fukunaga et al. (1995) identified several *Borrelia* species cultured from the midgut of *Ixodes* ticks feeding on black-faced bunting (*Emberiza spodocephala*), which was recognized as a new *Borrelia* species with remote similarity to Bbsl, relapsing-fever-type *Borrelia*, and *B. anserina*, named *Borrelia miyamotoi* [45].

Heylen et al. (2013) [46] described enzootic cycles of *Borrelia* in a system involving two resident songbirds (*P. major* and *C. caeruleus*) and a tick community consisting of three species (*I. arboricola*, *I. frontalis*, and *I. ricinus*). Of the 640 ticks screened for *Borrelia*, 227 were infected (35.5%), with altogether 7 strains of *Borrelia*, including Bbsl and relapsing-fever-type *Borrelia* [46].

Heylen et al. (2017) [47] published the results of a two-year study in which they collected ticks from 375 songbirds in the Netherlands and Belgium and screened the 671 ticks for infections. Bbsl DNA was found in 34% of the ticks, where 47% of the DNA was from *B. garinii*, and 16% from *B. afzelii* [47]. Heylen et al. [48] also published their results in the same year, from an experiment where they tried to infect birds with infected ticks. *Borrelia*-infected ticks were collected from songbirds. The generalist parasite *I. ricinus* and the ornithophile *I. frontalis* nymphs were attached to pathogen-naive birds, but the birds only started to selectively amplify the two avian species (*B. turdi* and *B. valaisiana*) [48].

### 2.3. Human-Pathogenic Borrelia in Birds, Reservoir Competence, and Tissue Tropism

*The next step toward demonstrating a more significant epidemiological role in human infections is the presence of human-pathogenic Borrelia species in birds’ blood and other tissues.*

In 1984 and with further confirmation in 1986, Anderson et al. [37,38] first isolated *B. burgdorferi* (not *B. anserina*) from a bird, the liver of a passerine bird, *Catharus fuscescens* (veery), and from larval *Ixodes dammini* (tick) feeding on *Pheucticus ludovicianus* (rose-breasted grosbeak) and *Geothlypis trichas* (common yellowthroat), by indirect immunofluorescence. Also, this is the first mention of *B. anserina* cultivation in BSK medium [37,38].

Mather et al. (1989) [49] performed the first tick xenodiagnosis. It aimed to show that an animal species can not only become infected with tick-borne Lyme disease, but that a tick that comes into contact with an infected animal and is not infected with *Borrelia* can also become infected. This is concrete evidence for the different animal species becoming distributors or “reservoirs” [49].

Despite this, Mather et al. (1989) [49] showed that the catbird (*Dumetella carolinensis*) cannot transmit a Bbsl infection to ticks parasitizing it, whereas mice can under the same conditions. Nevertheless, spirochetes were detected in ticks collected from two Carolina wrens (*Thryothorus ludovicianus*) and a common yellowthroat (*Geothlypis trichas*), but the infections were not proven to have originated in the birds [49]. This raised the question of whether the transmission of the infection depends on the bird species and the experimental conditions.

The proof was given by McLean et al. (1993) [50], who isolated a spirochete with a plasmid and protein profile similar to *B. burgdorferi* and not resembling *B. anserina* from 1 out of 39 passerine birds, a song sparrow (*Melospiza melodia*). The isolate could infect white-footed mice (*Peromyscus leucopus*) and golden hamsters (*Mesocricetus auratus*) [50]. Thus, a potentially human-pathogenic infection, originating in birds, could be transmitted to small rodents that are typical members of the enzootic cycle of human-pathogenic *Borrelia*.

Stafford et al. (1995) [51] further elaborated the conditions of reservoir competence in birds when they carried out a field study around Lyme, CT—the town where an outbreak of Lyme arthritis led to the eventual discovery of the pathogen *Borrelia burgdorferi*. Between 1989 and 1991, more than 5000 birds were captured and then the ticks collected from them were tested for *B. burgdorferi* using an IFA test. Here, 15.2% of birds were infested with *Ixodes scapularis*. Infection rates with *B. burgdorferi* varied by tick species, developmental stage, and host species. They concluded that “Spirochetemia in most avian hosts may be short and only certain species with concurrent infestations of nymphs and larvae may function effectively as reservoirs” [51].

Reservoir competence was proven by Miyamoto et al. (1997) [52], who first isolated *Borrelia* from migratory birds in Japan. Spirochetes were found in 4 of 11 brown-headed thrush (*T. chrysolaus*), and the species was identified as *Borrelia garinii*. This was considered proof that migratory birds are reservoirs for the transmission of the Lyme disease spirochetes in Japan [52].

*Further research was needed to determine which bird species are the best reservoirs for Borrelia and which Borrelia species are most suitable for transmission by birds—this was answered the following year.*

The exact process of transmission of natural and experimental human-pathogenic *Borrelia* infections was further investigated by Kurtenbach et al. (1998) [53], who made the first xenodiagnosis in birds in England. Pheasants infected via ticks were much more likely to transmit the infection than those infected with syringes (23% and 5%, respectively), but both groups were able to spread it for a time period of over three months after infection [53]. Kurtenbach, with other colleagues, published data in the same year showing that while pheasants were able to transmit *B. garinii* and/or *B. valaisiana* to non-infected xenodiagnostic ticks (and no other species of *Borrelia*), *B. burgdorferi s.s.* were only transmitted by rodents. This confirms that birds selectively amplify the two *Borrelia* species [54].

Gern et al. (1998) [55] reviewed European rodents and birds as hosts responsible for the spread of ticks, and with them Lyme disease. Nineteen new avian species can be considered as possible reservoir hosts for *B. burgdorferi* s.1., and there is strong evidence for sixteen bird species [55].

Heylen et al. (2014) confirmed that a European songbird (*Parus major*) can selectively amplify those *Borrelia* species that are common in birds (*B. garinii* and *B. valaisiana*) and not others (*B. afzelii*, *B. burgdorferi s.s*, and *B. spielmanii*) when infected via *Ixodes ricinus* [56].

Gylfe et al. (2000) also demonstrated that migratory birds can carry Bbsl as a latent infection for several months, and the infection can be reactivated and passed on to ticks [57].

Bózsik et al. (2024) published evidence of direct investigation by dark-field microscopy and an immunofluorescence microscopy method, of live Bbsl and *B. anserina* in the concentrated blood samples of wild birds: white stork (*Ciconia ciconia*), white-tailed eagle (*Haliaeetus albicilla*), and Eastern imperial eagle (*Aquila heliaca*) [17], simplifying how the presence of infection can be proven.

Jordan et al. (2009) [58] further elaborated on the bird–tick–*Borrelia* interaction by showing that tick species and *Borrelia* infections differed along the migratory routes of birds compared with those away from the routes. They demonstrated the presence of several *Borrelia* species in migratory birds and non-migratory wild turkeys, suggesting that migratory birds may play a role in the spread of Lyme disease spirochetes. Of the total 389 avian blood samples, 91 were positive for *Borrelia* spp. Among migratory birds and turkeys tested near migration routes, *B. burgdorferi* predominated. Among turkeys further away from flyways, *B. lonestari* was more common [58].

*After demonstrating reservoir competence, further investigations were undertaken to determine where human-pathogenic Borrelia reside in birds.*

Wagemakers et al. (2017) found that the spleens of 2 birds (*Carduelis chloris* and *P. major*) out of 26 collected in the Netherlands were infected with *B. miyamotoi* [59].

Norte et al. (2020) [60] investigated the tissue tropism of natural and experimental Bbsl infection in blackbirds (*Turdus merula*) and great tits (*P.s major*) and concluded that out of various tissues collected in vivo and during necropsy, skin biopsy was the most characteristic source of *Borrelia* DNA for the infectivity status of the birds, as compared with the *Borrelia* DNA concentration in the xenodiagnostic ticks. Even the usability of the skin samples depends on the time since infection, the species, and the genetic variant of *Borrelia* [60].

### 2.4. Human-Pathogenic Borrelia in Birds, Affecting the Birds’ Health Status: Seroconversion, Reinfection, and Health Effects

*Bbsl was initially considered a transient spirochete that did not elicit a reaction in the bird reservoirs themselves. However, several studies demonstrate that these infections elicit an immune response and other health effects in birds, and, unlike an avian-pathogenic infection, the immune response does not prevent a subsequent infection.*

Isogai et al. (1994) [61] demonstrated the reservoir potential of Japanese quail (*Coturnix japonica*) for the human-pathogenic *B. garinii* in Japan, and the critical fact that the birds are not just asymptomatic carriers. The concept of this publication builds on the previous research that identified a *B. garinii* strain—one very similar to isolates from humans—from the *Ixodes persulcatus* in Japan—a tick that is probably the most opportunistic tick species and, therefore, parasitizes both humans and birds [62]. Isogai and colleagues infected quails subcutaneously, mimicking a tick bite, and found that *B. garinii* can survive in quails for at least the period of examination (56 days). The birds will seroconvert between 53 and 56 days post-infection and may show occasional symptoms [61].

Richter (2000) [63] found that American robins (*Turdus migratorius*) served as a reservoir for Lyme disease spirochetes—robins acquired infection and became infectious to almost all xenodiagnostic ticks. Although infectivity waned after two months and disappeared after six months, the robins remained susceptible to reinfection [63].

Büker et al. (2013) demonstrated that human-pathogenic Bbsl are not only transitory guests in birds of prey, but they also generate an immune response: increased antibody levels could be found using a modified indirect immunofluorescent test in 4 out of 29 birds of prey [64]. This paved the way for further research on the interaction between human-pathogenic *Borrelia* and their bird reservoirs.

Norte et al. (2013) [65] further clarified this important question—whether the health status of birds carrying ticks infected with Bbsl and serving as reservoirs for human infections is affected by the ticks—by testing for several health markers. Tick infestation caused a stress response and reduced body mass in *Turdus merula.* In contrast, plasma globulin concentration in first-year birds tended to be affected when the ticks were infected with *Borrelia*. The two adult birds carrying infected ticks had higher white blood cell counts and glucose levels [65].

### 2.5. Birds Carrying Borrelia-Infected Ticks, or Borrelia Infections, Transcontinentally

*Infestation of migratory birds by ticks that may carry human-pathogenic infections makes them at least a short-term epidemiological factor for the infections. Proof of infection in the blood and organs of migratory birds makes this impact over a longer distance and over a longer term.*

Olsen et al. (1995) [3] investigated the transhemispheric transmission of infected ticks and Bbsl infections that *Ixodes uriae* carries. An identical *B. garinii* genotype was identified in ticks collected from the Northern or Southern hemisphere, proving the potential transmission [3]. Olsen, with other colleagues, also examined a total of 22,998 migratory birds in Scandinavia. The presence of Bbsl (primarily *B. garinii*) was proven by an immunofluorescence assay of tick larvae and DNA amplification by PCR on all ticks [66], proving the trans-European distribution of infected ticks.

Gylfe et al. (1999) [67] identified *B. garinii* DNA from 2 out of 102 puffins (*Fratercula arctica*) as reservoirs, and 3 out of 81 ELISA and Western blot tests were positive, in the Faeroe Islands. These medium-sized birds, although themselves mainly migrating smaller distances than some large-bodied birds, connect these islands to Northwestern Europe and North America [67].

When Younsi et al. (2005) compared the genome of the North-African/Portuguese Bbsl species *Borrelia lusitaniae* collected from the two regions, they found a moderate diversity in the whole genome, as well as in the ospA gene sequences [68]. It was reasonable to suggest that there was a direct connection between the two continents, with birds as a possible carrier/reservoir. Poupon et al. (2006) concluded that although *B. lusitaniae* is mainly found in lizards, birds may serve as a reservoir [69]. Cirkovic et al. (2024) [70] used multilocus sequence typing (MLST) and confirmed at least two introductions of *B*. *lusitaniae* from Europe to North Africa. Here again, birds were suspected, due to their migration routes, to be the link on the route: mainland Europe–(Spain)/Portugal–North Africa [70]. In a review article, Doss et al. (2024) confirmed that the distribution of Bbsl in Africa is not fully known, but the enzootic cycle is closely related to migratory birds, ticks of the *Ixodes* and *Hyalomma* genera, and lizards as possible reservoirs [71].

Schramm et al. (2014) reported the southernmost occurrence of Bbsl DNA in a seabird, proving also the serological reaction in tick-infested king penguins (*Aptenodytes patagonicus halli*) [72].

Norte et al. (2020) [73] examined 2308 ticks from 843 passerine birds in 11 European countries. Here, 37% of the 656 ixodid ticks were infected with Bbsl, mainly with *B. garinii* (60.7%), whereas the genetic variants of *B. garinii* showed no geographical structuring within Europe [73].

### 2.6. Summary Works on Borrelia–Bird Interactions

Hovind-Hougen (1995) provided a detailed morphological description of *B. anserina*, citing key literature from the previous decades and comparing it to the morphology of different spirochetes [74].

Humair (2002) [40] in the article “Birds and *Borrelia*” summarized evidence for the reservoir competence of birds for various species of *Borrelia*, as of the date of issuance. Birds may contribute to the maintenance of Bbsl through two distinct enzootic cycles: the terrestrial and marine cycles. In Europe, *B. garinii* is the main strain carried by birds, whereas in North America, *B. burgdorferi* sensu stricto (Bbss). Besides maintaining the local infections, migrating passerines and seabirds may contribute to the spread of *Borrelia* and of infected ticks [40].

Benskin et al. (2009) [75] described how migratory birds can serve as important transmitters of infected ticks and reservoirs of *Borrelia* infections. Ticks attach to their hosts for 24 to 48 h while they feed on blood. This duration provides migrating birds with sufficient time to cover large distances before the ticks finish feeding and detach, potentially introducing infectious ticks into new geographic regions. There is documented evidence of transhemispheric movement of spirochete-infected ticks by seabirds, highlighting the ability of wild birds to transport infected ticks over extensive distances. Even if the overall ectoparasite load is low, the number of birds carrying tick vectors can significantly impact local tick populations, thereby influencing disease dynamics. Additionally, birds can harbor infections within their bloodstream, which can subsequently be transmitted to local tick populations at different locations [75].

Hasle (2013) collected literature on the transport of ixodid ticks and tick-borne pathogens by migratory birds, especially *Turdus* spp., being a reservoir for *B. garinii* [76].

Ivanova Aleksandrova (2020) [77] collected the most important literature references that indicate the reservoir competence and the role of migratory birds in the spreading of Lyme borreliosis, especially emphasizing the fact that birds spread ticks infected with, and infect ticks feeding on them mostly with, *B. garinii*. This is an essential fact that underscores the high number of cases of Lyme neuroborreliosis (or Lyme borreliosis with neurological symptoms) across Europe that are attributable to a *B. garinii* infection, as opposed to Lyme disease with other leading symptoms generally caused by other *Borrelia* species, such as *B. afzelii*, where, for example, small rodents have a reservoir competence [77].

## 3. Conclusions

Avian borreliosis has historically contributed to the development of microbiology, especially due to the special properties of *B. anserina*. This spirochete is easy to recognize and abundantly present in blood samples. In recent decades, the identification of Bbsl as the causative agent of Lyme disease revitalized *Borrelia* research; however, more advanced techniques were needed to reliably detect the scarce spirochetes in blood and tissue samples.

Today, bird species are known to carry infected ticks, harbor human-pathogenic *Borrelia* in their blood and organs, and selectively amplify specific species, particularly *B. garinii*. While it was once believed that these human-pathogenic species existed in birds without causing symptoms, current evidence indicates that they can induce an immune response, cause symptoms, be reactivated, and like in humans, immunity does not prevent reinfection.

Wild migratory birds can thus play a role in the spread of the most important human–bird infection, *B. garinii*, which is neurotropic in humans and currently present mainly in Eurasia.

Future research is expected to reveal the importance of migratory wild birds in the dissemination of *B. garinii*. Additionally, the health effects of human-pathogenic and other emerging *Borrelia* species in birds may be elucidated.

## Data Availability

No new data were created or analyzed in this study. Data sharing is not applicable to this article.
